# Seminal Fluid Affects Sperm Viability in a Cricket

**DOI:** 10.1371/journal.pone.0017975

**Published:** 2011-03-24

**Authors:** Leigh W. Simmons, Maxine Beveridge

**Affiliations:** School of Animal Biology, Centre for Evolutionary Biology, The University of Western Australia, Crawley, Australia; Aarhus University, Denmark

## Abstract

Recent studies have suggested that males may vary the quality of their ejaculates in response to sperm competition, although the mechanisms by which they do so remain unclear. The viability of sperm is an important aspect of ejaculate quality that determines competitive fertilization success in the field cricket *Teleogryllus oceanicus*. Using *in vitro* mixtures of sperm and seminal fluid from pairs of male crickets, we show that seminal fluid can affect the viability of sperm in this species. We found that males who invest greatly in the viability of their own sperm can enhance the viability of rival sperm, providing the opportunity for males to exploit the investments in sperm competition made by their rivals. Transitive effects of seminal fluids across the ejaculates of different males are expected to have important implications for the dynamics of male investments in sperm competition.

## Introduction

Sperm competition theory predicts that multiple mating by females should favour the evolution of increased male expenditure on the ejaculate as males compete to fertilize available ova [1]. Moreover, because ejaculates are costly to produce, selection is predicted to favour phenotypic plasticity in expenditure such that males invest more in their ejaculate when they encounter females who are more likely to mate with additional males [1]. Consistent with theory, among species, positive evolutionary associations have been found between testes mass and estimates of the strength of selection generated by sperm competition [2], [3], and within species, exposing males to an elevated risk of sperm competition can result in them transferring more sperm at copulation [4].

Empirical studies have focussed primarily on the effects of sperm competition on the numbers of sperm ejaculated, with little attention being paid to variation in ejaculate quality [5]. However, there is now some evidence to suggest that males may also be able to make strategic adjustments to the quality of their ejaculates. Thus, in fish [6], [7], fowl [8], and humans [9], males have been shown to be capable of making rapid adjustments to the swimming velocity of sperm contained within their ejaculates, in response to social status, the attractiveness of their mating partner, or the perceived risk that they will face sperm competition. Male crickets, *Teleogryllus oceanicus*, are capable of making adjustments to the quality of their sperm in response to both the risk and intensity of sperm competition; when males perceive the presence of a potential rival they produce ejaculates with a greater proportion of viable sperm than when isolated from other males [10]. Moreover, the males of this species also appear to recognise individually distinct cuticular hydrocarbon residues left on a female after mating, making fine-grained assessments of the actual number of males in competition and reducing their expenditure on sperm viability accordingly [11], [12]. The mechanism by which males make strategic adjustments in sperm quality is unknown, but recent work on fowl suggests that adjustments in seminal fluid quality may be an important modulator of sperm performance [13].

Seminal fluid is known to contain important biologically active compounds that affect male fertility [14]. The role of seminal fluid proteins in influencing female fecundity is also well documented [2], particularly in *Drosophila melanogaster* where seminal fluid proteins reduce female receptivity to further matings, and increase rates of oviposition at a cost to female lifespan [15], [16]. However, with the notable exception of *D. melanogaster* [17], [18], [19] the function of seminal fluid in the context of competitive fertilization success is less well studied. Cameron et al.'s [20] theoretical analysis predicts that selection should favour phenotypic plasticity in male expenditure on both sperm and non-sperm components of the ejaculate that affect its competitive weight, but the predictions from their models depend critically on which component of the ejaculate, sperm or seminal fluid, influences its competitive weight. These findings call for empirical studies that examine how sperm and non-sperm components of the ejaculate influence its competitiveness. For *T. oceanicus* we know that sperm viability, rather than sperm numbers per se, is the principal determinant of competitive fertilization success [21]. Here, by separating sperm from seminal fluid components of the ejaculate, and conducting reciprocal recombination within and among males, we show how seminal fluid affects the viability of self and rival sperm.

## Methods

**Figure 1:**
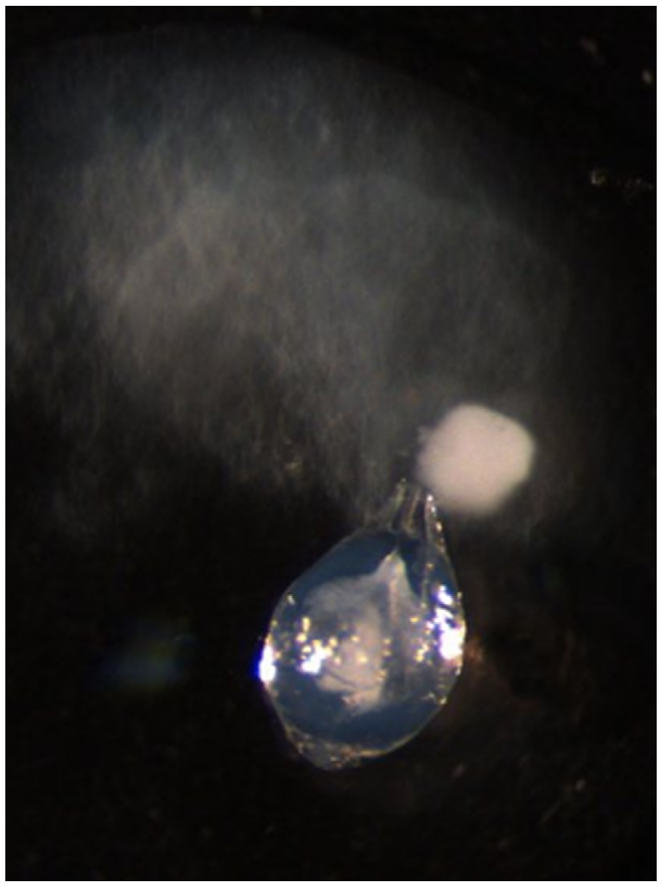
An evacuated spermatophore. The seminal fluid (white) discharges only after all sperm (grey) have left the spermatophore.

Male crickets were derived from an outbred laboratory culture, and isolated as final instar nymphs in individual boxes supplied with cat chow and a cotton plugged tube containing water. Crickets were maintained in a constant temperature room (25°C with a 12∶12 hour light∶dark cycle), and monitored daily. Fourteen days after they moulted to adulthood, 42 unrelated crickets were paired at random to provide 21 experimental pairs. A spermatophore was removed from the genital pouch of each male and placed onto a separate cavity slide in 20 µl of Beadle saline (128.3 mM NaCl, 4.7 mM KCl, and 23 mM CaCl_2_), the evacuating tube was cut from the neck of the spermatophore with fine scissors, and evacuation observed under a binocular microscope. Under these conditions sperm were discharged from the spermatophore over a period of ∼20 min, following which there was a brief period (10–20 sec), before the seminal fluid was discharged (see Fig. 1). After sperm ceased to be discharged, they were immediately removed along with 10 µl of the Beadle saline, and placed into a clean 0.5 ml eppendorf tube. Once the seminal fluid had discharged, it was likewise placed into a clean eppendorf tube. Reciprocal combinations of sperm and seminal fluid were then made for each pair of males. Thus 2.5 µl of sperm from each male in a pair was mixed with either 2.5 µl of his own seminal fluid or 2.5 µl of the other male's seminal fluid. The viability of sperm in each of the four samples was then assessed using a live-dead assay (Molecular Probes). Each 5 µl aliquot of sperm and seminal fluid was mixed with 5 µl 1∶50 diluted 1 mM SYBER-14 and the sample incubated in the dark for 10 min, before adding 2 µl propidium iodide and incubating for a further 10 min. Samples were viewed under a florescence microscope and the number of live (stained green by SYBR-14) and dead (stained red by propidium iodide) sperm in the first 500 sperm counted was used to calculate the proportion of live sperm in the sample.

## Results

**Figure 2:**
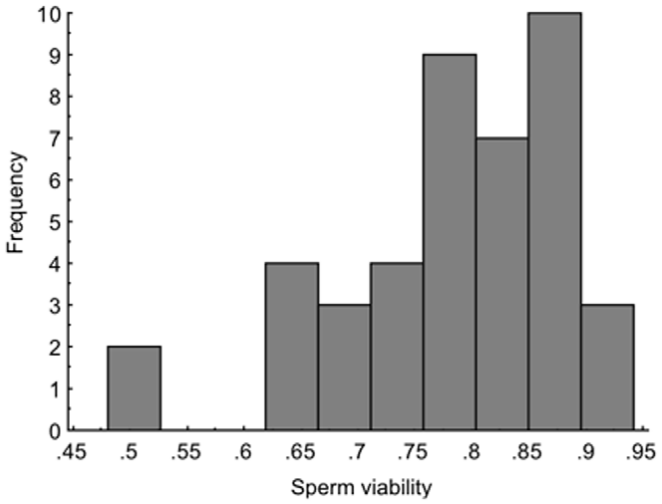
Frequency distribution of sperm viability for the males used in this study. The data are for sperm mixed with the male's own seminal fluid.

**Figure 3:**
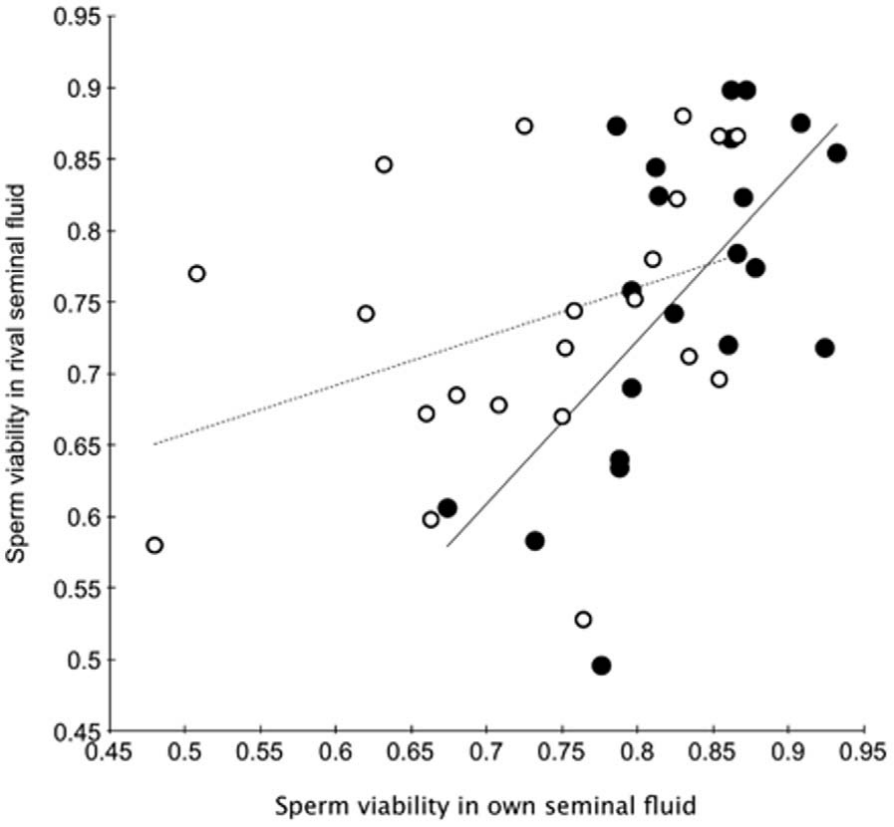
Relationship between viability of sperm in own versus rival seminal fluid. Closed (open) symbols show the effects of the seminal fluid from males of a pair with relatively high (low) sperm viability on their lower (higher) viability rival.

Across all males, when mixed in the male's own seminal fluid the mean ±SE viability of sperm was 0.78±0.02, and ranged from 0.48–0.93 (Fig. 2). For each male pair we were able to identify the male with the higher (H) and lower (L) sperm viability when his sperm were mixed with his own seminal fluid; on average males within pairs differed significantly in their sperm viability (higher males 0.83±0.01, lower males 0.73±0.02; matched-pairs t = 5.23, df 20, P<0.0001). The difference in the viabilities of sperm between males of a pair when in their own seminal fluid ranged from as little as 0.6% to as much as 31%. Thus some males were closely matched while others differed greatly. We contrasted the viability of sperm in own versus rival seminal fluid using a regression analysis approach. If the seminal fluid of rivals had no impact on a male's sperm viability, the viability of his sperm should be the same whether mixed with his own or rival seminal fluid. Thus, under the null hypothesis regressing sperm viability in own seminal fluid on sperm viability in rival seminal fluid is expected to yield a slope of 1.0 and an intercept of zero. When sperm from the male with a high viability ejaculate were combined with the seminal fluid from his rival with a relatively low viability ejaculate, the viability of his sperm was unaffected; the slope did not differ from 1.0 (1.14±0.32, t = 0.45, df 19, P = 0.658) and the intercept did not differ from zero (−0.19±0.26, t = 0.71, P = 0.484) (closed symbols in Fig. 3). In contrast, for the relatively low viability male of a pair, the viability of his sperm was enhanced when mixed with seminal fluid from his rival with high sperm viability; the slope was significantly less than 1.0 (0.34±0.19, t = 3.39, P = 0.003) and the intercept was significantly greater than zero (0.49±0.14, t = 3.38, P = 0.003) (open symbols in Fig. 3). The data in Figure 3 show that the males who benefited from rival seminal fluid were those whose sperm had very low viability when in their own seminal fluid.

## Discussion

We show that seminal fluid influences the viability of sperm in the cricket *T. oceanicus*. Sperm viability in this species is a key factor determining competitive fertilization success [21], so that seminal fluid is likely to be under significant postcopulatory sexual selection. Recent molecular studies of cricket accessory gland genes have identified 30 genes predicted to encode seminal fluid proteins, a significant proportion of which appear to be under positive selection [22], [23]. Assuming that the patterns we have observed *in vitro* extend to *in vivo* interactions among ejaculates, our study suggests that one important function of seminal fluid proteins may lie in sperm competition.

Sperm incapacitation has long been suggested as a potential mechanism of sperm competition, but empirical evidence for incapacitation has generally been equivocal [24], [25], [26]. Two recent studies have reported opposite effects of seminal fluid on rival sperm viability. In *D. melanogaster*, seminal fluid was found to increase the viability of sperm, irrespective of its source [26]. Thus, the viability of sperm was increased after the addition of own and rival seminal fluid. In contrast, in multiple mating social bees and ants, own seminal fluid was found to increase sperm viability, while rival seminal fluid was found to decrease sperm viability [27]. Our study shows that the effects of seminal fluid on sperm viability are likely to depend strongly on ejaculate-by-ejaculate interactions [28], [29]. Consistent with the work on *D. melanogaster*, we show that the viability, and thus competitive weight of a male's sperm, can be increased by the seminal fluid of his rival. Thus, seminal fluid produced by males with relatively high sperm viability was found to increase the viability of sperm in the ejaculate of an inferior competitor. However, males who produced ejaculates with high sperm viability were unaffected by the seminal fluid of their rivals. Thus, different effects can be observed, dependent on the potency of donor and recipient seminal fluids.

The considerable variation in seminal fluid potency that we observe could provide an avenue by which males can exploit the seminal fluid investments of their rivals [30]. For example, if males mating to unmated females invest heavily in seminal fluid to enhance the viability of their sperm, subsequent males could reap the benefits of their rivals past investment while saving their own seminal fluids for future matings. Although male *T. oceanicus* exhibit intrinsic genetically based differences in sperm viability [31], they also show phenotypic plasticity in this trait, reducing their investment in sperm viability when mating with females that have already received sperm and seminal fluids from previous males [10], [12]. Strategic adjustments in seminal fluid investments are predicted by recent theoretical models that incorporate both sperm and non-sperm components of ejaculate investment [20], and our data suggest that adjustments in seminal fluid potency could underlie the strategic adjustments in sperm viability observed in this species [10], [12]. Indeed, recent work with *D. melanogaster* has revealed that males can adjust the protein composition of their seminal fluids in response to perceived risk and intensity of sperm competition [32].

The effects of seminal fluid on sperm viability that we document here are remarkably similar to the effects of seminal fluid on the viability of own and rival embryos previously documented in this species. Male *T. oceanicus* that impart high viability to their own embryos also impart high viability to embryos sired by their rivals [33]. Paternal effects on embryo viability have been found to be genetically correlated with a male's investment into his accessory glands [34]. It may be that different seminal fluid compounds act independently on sperm and embryos to impart viability. Alternatively, seminal fluid compounds may affect qualitative differences in sperm, other than their viability, that impact developing embryos. The DNA integrity of sperm can be protected by seminal fluid proteins [35], [36], which in turn can determine the viability of embryos sired [37], [38], [39]. Identifying the seminal fluid compounds responsible for effects on sperm and embryo viability in *T. oceanicus* will allow us to explore more fully the evolutionary significance of this biologically important fluid.
